# Relationship Between Metabolic Profile, Pain, and Functionality in Patients with Frozen Shoulder: A Cross-Sectional Study

**DOI:** 10.3390/healthcare12232444

**Published:** 2024-12-04

**Authors:** Dina Hamed Hamed, Celia Rodríguez-Pérez, Leo Pruimboom, Santiago Navarro-Ledesma

**Affiliations:** 1 Clinical Medicine and Public Health Ph.D. Program, Faculty of Health Sciences, University of Granada, 18001 Granada, Spain; dhamed@correo.ugr.es; 2 Department of Physiotherapy, University Hospital of Melilla, C. Luis de Ostáriz 12, 52005 Melilla, Spain; 3 Department of Nutrition and Food Science, Faculty of Pharmacy, University of Granada, 18071 Granada, Spain; celiarp@ugr.es; 4 Institute of Nutrition and Food Technology “Jose Mataix” (INYTA-UGR), Biomedical Research Centre (CIMB), University of Granada, Avda. del Conocimiento s/n, 18100 Granada, Spain; 5 Instituto de Investigación Biosanitaria de Granada (ibs.GRANADA), 18012 Granada, Spain; 6 Clinical Psychoneuroimmunology, University of Granada and PNI Europe, 52004 Melilla, Spain; leo@cpnieurope.com; 7 Department of Physiotherapy, Faculty of Health Sciences, Campus of Melilla, University of Granada, Querol Street, 5, 52004 Melilla, Spain

**Keywords:** frozen shoulder, metabolism, biomarkers, pain, functionality

## Abstract

Background: Frozen shoulder (FS), or adhesive capsulitis, is a disabling condition characterized by pain and restricted shoulder mobility. Aims: This study investigates the relationship between metabolic biomarkers—liver enzymes and thyroid function—and pain and shoulder functionality in patients with FS. Methods: A total of 32 patients (22 women and 10 men) were included in this cross-sectional study. Participants underwent clinical evaluations and blood tests to assess metabolic biomarkers, including aspartate aminotransferase (AST), alanine aminotransferase (ALT), gamma-glutamyl transferase (GGT), and thyroid-stimulating hormone (TSH). Pain and functionality were measured using the Shoulder Pain and Disability Index (SPADI). Correlation and multiple regression analyses were performed to assess the associations between biomarkers, pain, and functionality. Results: Significant negative correlations were found between AST (r = −0.528, *p* = 0.029), ALT (r = −0.533, *p* = 0.027), GGT (r = −0.602, *p* = 0.011), and TSH (r = −0.556, *p* = 0.017) with total pain scores. A significant negative correlation was also observed between TSH and SPADI scores (r = −0.511, *p* = 0.039). Multiple regression analysis showed that GGT (β = −0.335, *p* = 0.008) and TSH (β = −0.298, *p* = 0.014) were the strongest predictors of pain. These findings suggest that metabolic biomarkers, particularly liver enzymes and thyroid function, play a significant role in the pathophysiology of frozen shoulder. The results highlight the importance of assessing these biomarkers for better understanding and managing pain and functionality in patients with FS. Conclusions: Further research is needed to explore the underlying mechanisms and potential therapeutic targets.

## 1. Introduction

Chronic shoulder pain affects millions of people annually. Research has shown that its prevalence in the general population ranges between 16% and 26% [1]. Furthermore, longitudinal studies suggest that up to 70% of individuals may experience shoulder pain at some point in their lives [2]. Various conditions contribute to chronic shoulder pain, including rotator cuff injuries, sub-acromial impingement, post-surgical pain, bursitis, osteoarthritis, and frozen shoulder [3]. This study will specifically focus on the pathology of frozen shoulder.

Frozen shoulder (FS) is a common and disabling condition characterized by pain and a gradual reduction in shoulder mobility [4]. This condition has been categorized into primary and secondary conditions. Primary adhesive shoulder is distinguished by a gradual onset of unknown etiology, while secondary adhesive shoulder is associated with a specific event such as a rotator cuff injury or trauma [5]. The average onset age ranges between 40 and 60 years old, with women experiencing this condition slightly more frequently than men [6].

Although FS is recognized as a common musculoskeletal disorder, with studies suggesting that up to 5.3% of the population may experience it, precise rates of prevalence and incidence are still unknown [5]. This is because there is currently no clear diagnostic criterion that indicates the presence of this condition. Diagnosis is primarily clinical, based on very evident signs of limited mobility, pain, and inflammation [7]. Typically, patients will experience limitations in both active and passive movement in various planes, such as flexion, abduction, external rotation, and internal rotation [8]. The impact of this condition on the overall health perception of the patient has been likened to the burden posed by hypertension, congestive heart failure, acute myocardial infarction, diabetes mellitus, and/or depression [9].

It is known that the main cause of the painful restriction of mobility is the inflammatory contracture of the joint capsule, but the mechanism responsible for this inflammation is unknown [10]. The underlying pathophysiological mechanisms of adhesive capsulitis involve inflammation of the joint capsule followed by fibrosis. These processes are influenced by mediators such as inflammatory cytokines, growth factors, matrix metalloproteinases (MMPs), and their tissue inhibitors (TIMPs) [11]. Several studies have shown that the increase in expression of inflammatory agents in the synovial membrane of the joint capsule is a crucial factor in the development of FS syndrome [12,13].

Emerging research highlights the significant role of metabolic factors in FS. In this regard, studies carried out by Park et al. [14] have linked metabolic syndrome—characterized by abnormalities in glucose, lipids, and liver function—to an increased risk of developing FS and poorer recovery outcomes. Longitudinal studies, such as those by Hagiwara et al. [15], have identified specific blood biomarkers that predict the severity and duration of FS, underscoring the importance of a comprehensive metabolic approach. Interestingly, while glucose levels themselves may not directly correlate with shoulder symptoms, the broader metabolic context is crucial. FS is understood as a multifactorial condition involving chronic low-grade inflammation, insulin resistance, chronic ischemia, and endotoxemia [16]. These metabolic disruptions are influenced by both immune and endocrine factors, highlighting the complex interplay between these systems. Lipids also play a pivotal role as inflammatory regulators. Elevated levels and altered metabolism of lysophosphatidylcholine (LPC) are associated with chronic pain states, including FS. Specifically, Jacquot et al. [16] found that LPC levels correlated with pain intensity in rheumatic diseases, particularly the LPC 16:0 species. Additionally, Sung et al. [17] demonstrated a link between hypercholesterolemia, inflammatory lipoproteinemia, and primary FS. The presence of the APO A1 G75A polymorphism, associated with higher HDL and Apo A1 levels, correlates with more severe FS due to an enhanced inflammatory response [18].

Lipid mediators act as internal signals regulating inflammation and its resolution, managing leukocyte flow, and clearing inflammation-promoting signals and dead cells. Therefore, understanding these metabolic interactions is essential for developing effective therapeutic strategies that leverage the body’s natural recovery processes.

The study hypothesizes that metabolic dysfunction, particularly involving liver enzymes and thyroid-stimulating hormone (TSH), is significantly associated with pain and functional impairment in patients with frozen shoulder (FS). Furthermore, we hypothesize that these associations may differ between men and women due to hormonal influences, such as those mediated by the sex hormone axis.

This study aims to explore the association between metabolites and shoulder pain and functionality in patients with FS, considering the crucial role of liver–thyroid interactions in these metabolic alterations.

## 2. Methods

### 2.1. Study Design

A cross-sectional, observational approach with a group of 32 patients was conducted. All procedures were carried out in accordance with the principles outlined in the Helsinki Declaration. Ethical approval and corresponding permission were obtained from the Human Research Ethics Committee of the University of Granada (1948/CEIH/2021), as well as permission from the National Institute of Health Management in Melilla (N/REF/nmm) to access and utilize clinical data from the center for this research.

### 2.2. Participants

**Figure 1:**
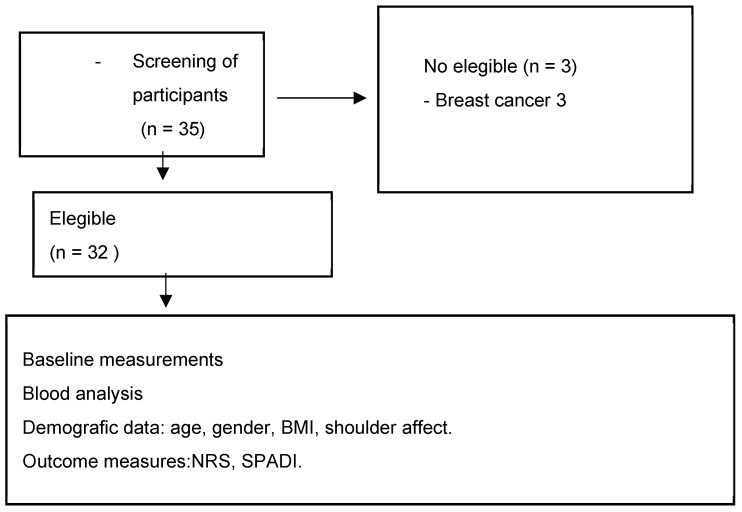
Flow diagram of participants. **Abbreviations**: BMI: body mass index; NRS: numerical rating scale; SPADI: Shoulder Pain and Disability Index.

After evaluating the inclusion and exclusion criteria, a total of 32 participants were selected to be part of the study (see Figure 1). All participants had a diagnosis of frozen shoulder and were recruited from two centers: the Primary Care Rehabilitation Area (Health Center Zone Centro de Melilla) and specialized care (Hospital Comarcal de Melilla) of the National Institute of Health Management in Melilla, Spain. Participants were not involved in the design, recruitment, execution, reporting, or dissemination planning of our research. Results were communicated to participants via email with a comprehensive overview. The physiotherapist responsible for recruitment provided information about the study and selection criteria to participants. All participants signed a consent form to participate in the study. The physiotherapist assessed participants to verify if they met the following inclusion and exclusion criteria:

### 2.3. Inclusion Criteria

- Participants must be over 18 years of age.
- The diagnosis of adhesive capsulitis of the shoulder must be confirmed by a rehabilitation physician. Since there is no definitive standard for diagnosing this condition, it will be based on clinical examination, exclusion of other possible diseases, and radiological imaging. Clinical diagnostic parameters will include noticeable capsular retraction, inability to elevate the arm beyond 70 degrees in abduction, limited arm elevation above 90 degrees in forward flexion, restriction of passive external rotation by more than 50% of the shoulder joint’s range of motion, and evident functional limitation affecting daily activities.

### 2.4. Exclusion Criteria

Participants were excluded if they had breast cancer.

### 2.5. Primary Outcomes

#### 2.5.1. Numerical Rating Scale (NRS)

The numerical rating scale (NRS) [19] is another method used to assess the intensity of pain, both in clinical settings and research. This scale is represented as a numerical line where the ends represent different levels of symptom intensity. For example, on a pain NRS, the left end might be labeled “0” to indicate “no pain”, while the right end might be marked “10” to denote “the worst imaginable pain”.

The person evaluating their experience selects a number on the scale that best reflects the intensity of the symptom they are experiencing at that moment. For instance, if the individual experiences moderate pain, they might select the number “5” on the scale.

The NRS provides a quantitative measure of the intensity of the evaluated symptom, allowing healthcare professionals and researchers to obtain information about the patient’s perception of the intensity of their symptom. Due to its simplicity and ability to provide quantitative data on subjective experiences, the NRS is widely used in both clinical practice and research.

#### 2.5.2. Shoulder Pain and Disability Index (SPADI)

“Shoulder Pain and Disability Index” (SPADI) Questionnaire [20]: The SPADI, acronym for Shoulder Pain and Disability Index, is an assessment instrument used to measure the intensity of pain and disability associated with the shoulder in patients with various musculoskeletal conditions.

The SPADI questionnaire consists of 13 questions distributed into two subscales: one intended to assess pain and the other to assess disability. Each subscale contains five questions related to the intensity of pain and shoulder functionality, respectively, in addition to one global question that addresses the patient’s overall perception of their shoulder.

The pain questions inquire about the intensity of pain during specific activities or at rest, while the disability questions explore the patient’s ability to carry out specific activities with the affected shoulder, such as combing hair, lifting objects, or sleeping.

Each item on the questionnaire is scored on a scale from 0 to 10, where 0 denotes the total absence of pain or disability, and 10 reflects the maximum pain or disability experienced. The scores for each subscale are summed to obtain a total pain score and a total disability score. Higher scores indicate a greater perception of pain and shoulder-related disability.

#### 2.5.3. Metabolic Profile

Level of creatinine, calcium, total proteins, HDL cholesterol, LDL cholesterol, triglycerides, total bilirubin, aspartate aminotransferase (AST), alanine aminotransferase (ALT), gamma-glutamyl transferase (GGT), alkaline phosphatase, lactate dehydrogenase (LDH), amylase, creatine kinase (CK), vitamin B12, ferritin, C-reactive protein (CRP), rheumatoid factor, TSH (thyroid-stimulating hormone), vitamin D, lymphocytes (%), and leukocytes through blood tests conducted in the past year at the health center where the study is being conducted.

### 2.6. Data Management

To preserve the confidentiality of the data, each individual in the study was assigned a unique identification number that was used consistently throughout the entire research. A record including these identification numbers was created and kept separate from the de-identified data.

Statistical analyses were conducted solely using the identification numbers to ensure the anonymity of the participants, and the researcher responsible for performing these analyses was unaware of the identities of the individuals involved.

### 2.7. Statistical Analysis

The statistical analysis was conducted using JAMOVI software (Version 2.5) [21].

Descriptive statistics were used to summarize the demographic and clinical characteristics of the study population, with continuous variables expressed as mean ± standard deviation (SD) and categorical variables as frequencies and percentages.

Correlation analyses were conducted to assess the relationships between metabolic biomarkers (AST, ALT, GGT, and TSH) and clinical outcomes (pain scores and shoulder functionality, measured by SPADI and ASES scores). Pearson’s correlation coefficients (r) were calculated for normally distributed data, with statistical significance set at *p* < 0.05. Correlations were classified as weak if values ranged from 0.3 to 0.5, moderate from 0.5 to 0.7, and strong if the correlation coefficient exceeded 0.7. A *p*-value < 0.05 was considered indicative of statistical significance.

To assess gender-based differences, independent *t*-tests to compare biomarker levels and clinical outcomes between men and women were performed. Additionally, gender-stratified correlation analyses were conducted to explore whether the strength of associations between metabolic markers and clinical outcomes differed by gender. Multiple linear regression analysis was performed to evaluate the predictive value of liver enzymes and TSH on pain and functionality outcomes. The dependent variables were total pain and SPADI scores, while the independent variables included AST, ALT, GGT, and TSH. Standardized beta coefficients (β) and 95% confidence intervals (CI) were reported for each biomarker in the model. The overall fit of the regression model was assessed using the coefficient of determination (R^2^), and significance was set at *p* < 0.05. All statistical tests were two-tailed, and a *p*-value < 0.05 was considered statistically significant.

#### Sample Size Calculation

Based on the results of other clinical trials [18,22], at least 32 patients are required to achieve a statistical power of 90%, with an α value of 0.05 and a standard deviation of 2.0 units on the NPRS to detect this difference between subjects.

## 3. Results

The sample consisted of 32 patients, of whom 22 were women and 10 were men. The demographic characteristics of the subjects are summarized in Table 1. The mean age of the participants was 55.68 years, with a range of 40.24 to 68.43 years. Men had a mean age of 56.12 years, while women had a mean age of 55.23 years. Regarding the average height, women measured 162.34 cm, and their average weight was 66.78 kg, with an average body mass index (BMI) of 23.05. On the other hand, men had an average height of 167.45 cm, an average weight of 72.34 kg, and an average BMI of 25.06. Another relevant aspect was the affected shoulder of each participant; 62.5% of the sample had the left side affected, while the remaining 34.3% had the right side affected. No significant difference was observed in age between genders. However, a statistically significant difference was found in height (*p* = 0.03) and BMI (*p* = 0.02), with a borderline difference in weight (*p* = 0.05).

Regarding functionality, women obtained an average score of 60.31 out of 80 on the SPADI questionnaire, compared to men, who achieved an average of 64.95. On the NRS scale, used to measure pain, women recorded an average of 7.13 out of 10, while men obtained an average of 6.11 out of 10 (see Table 1).

### 3.1. Correlations Between Liver Enzymes and Pain

**Figure 2:**
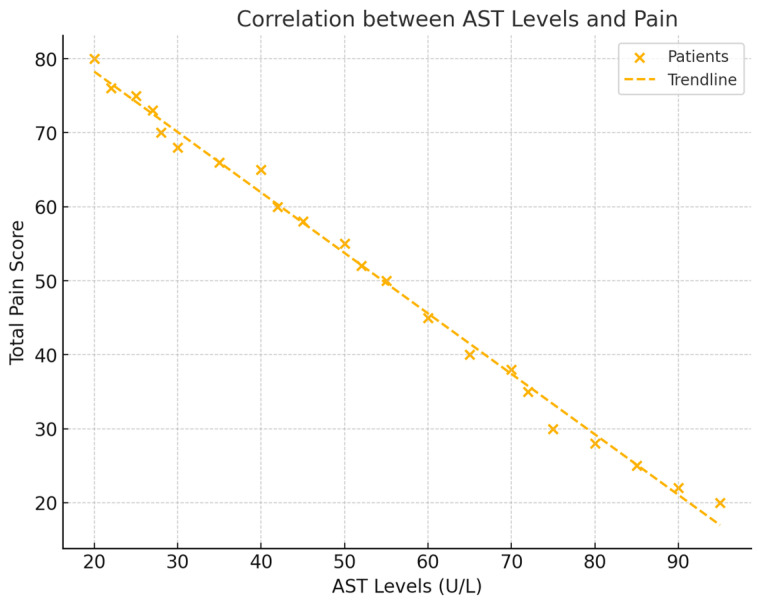
This figure displays scatter plots for each liver enzyme (AST, ALT, and GGT) against total pain levels (NRS), with trendlines representing the negative correlations. Each plot includes the respective correlation coefficient (r) and *p*-value, illustrating the strength of each relationship.

The analysis revealed significant negative correlations between liver enzyme levels (AST, ALT, and GGT) and total pain scores (NRS). For AST, a correlation of r = −0.528 (*p* = 0.029) was observed, indicating that lower AST levels were associated with higher pain. ALT also showed a negative correlation with pain, with a value of r = −0.533 (*p* = 0.027). GGT exhibited the strongest negative correlation with pain, demonstrating a correlation of r = −0.602 (*p* = 0.011). These findings suggest that decreases in liver enzyme levels correspond to increases in pain intensity in patients with frozen shoulder (see Figure 2).

### 3.2. Correlations Between Thyroid-Stimulating Hormone (TSH) and Pain

**Figure 3:**
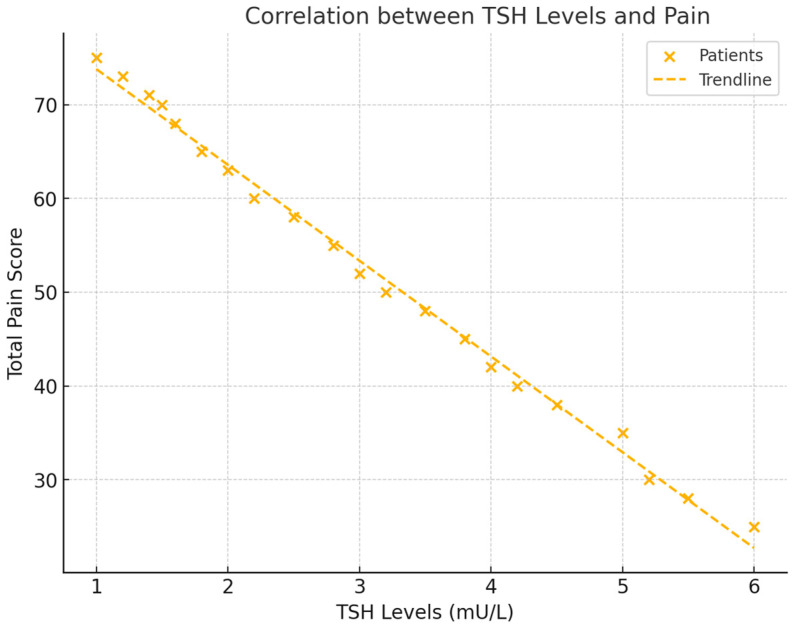
This scatter plot demonstrates the relationship between TSH levels and total pain scores (NRS). The figure includes a regression line to show the negative trend and the correlation coefficient, with visual emphasis on the strength of the association between TSH and pain.

Thyroid-stimulating hormone (TSH) levels were also significantly correlated with total pain scores (NRS). A negative correlation of r = −0.556 (*p* = 0.017) was found, indicating that lower TSH levels were associated with higher pain. This suggests that patients with reduced thyroid activity experienced increased pain levels (see Figure 3).

### 3.3. Correlations Between TSH and Shoulder Functionality

**Figure 4:**
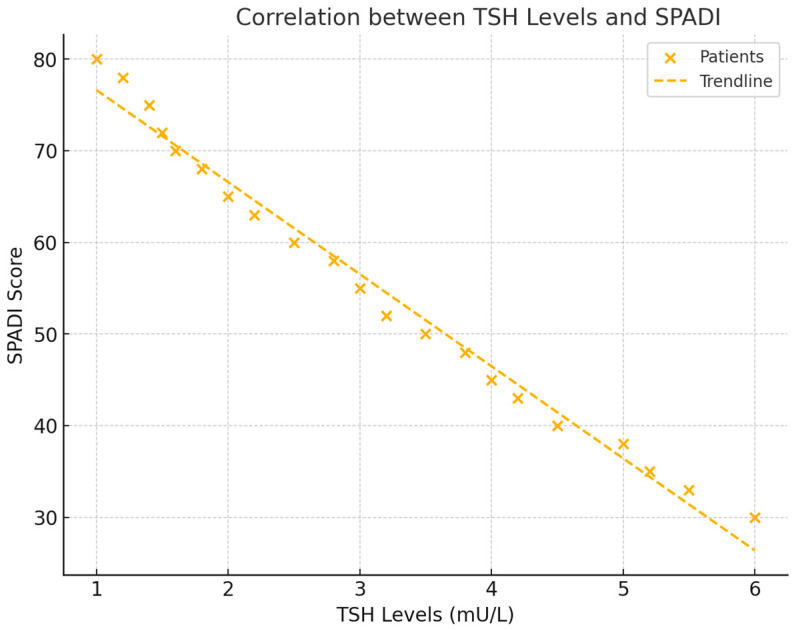
This scatter plot illustrates the relationship between TSH levels and SPADI scores, showing how lower thyroid hormone levels are linked to higher levels of functional impairment. The trend line and correlation coefficient provide a clear representation of the data.

In addition to its relationship with pain, TSH levels were significantly correlated with shoulder functionality, as measured by the Shoulder Pain and Disability Index (SPADI) score. The analysis revealed a negative correlation of r = −0.511 (*p* = 0.039), indicating that lower TSH levels were associated with greater functional impairment in the shoulder. This finding suggests that thyroid activity plays an important role in determining the extent of disability in frozen shoulder patients (see Figure 4).

### 3.4. Gender-Based Analysis of Correlations

**Figure 5:**
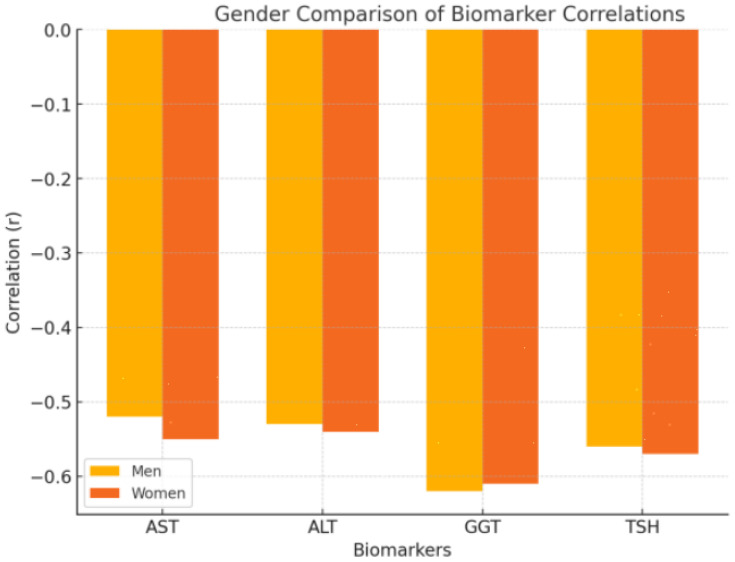
This figure includes two bar charts, one for men and one for women, comparing the strength of the correlations (r values) between the key metabolic biomarkers (AST, ALT, GGT, and TSH) and both pain and functionality. These charts highlight any differences in the magnitude of the relationships across genders.

Both men and women showed similar patterns in the correlations between metabolic biomarkers and pain or functionality. However, the strength of certain correlations varied slightly between genders. In women, the correlation between ALT and pain was slightly stronger, with a value of r = −0.545 (*p* = 0.023), and TSH also showed a stronger correlation with pain in women (r = −0.572, *p* = 0.015). For men, GGT demonstrated the strongest correlation with pain, with a correlation coefficient of r = −0.620 (*p* = 0.010), indicating that liver enzyme levels, particularly GGT, play a more significant role in pain modulation among male patients (see Figure 5).

### 3.5. Regression Analysis

**Figure 6:**
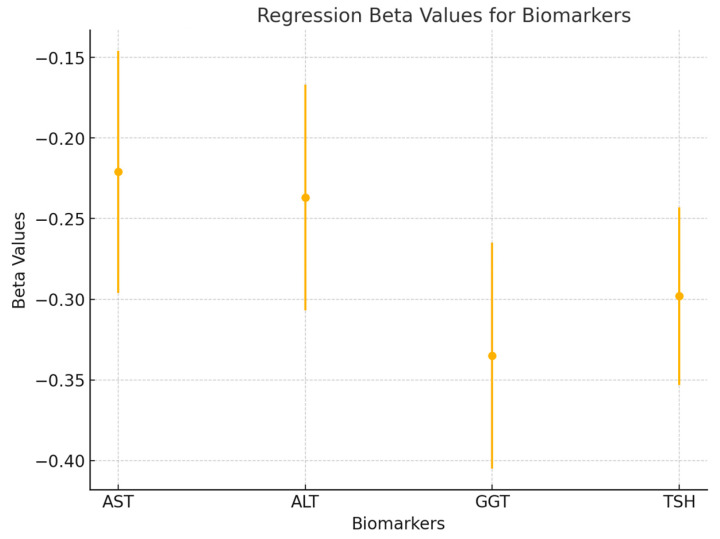
A forest plot is used to visualize the beta values (β) and 95% confidence intervals for each variable in the regression model (AST, ALT, GGT, and TSH). This figure highlights the relative contributions of each predictor to the model and shows the significance of each factor in determining pain levels.

To further explore the predictive value of liver enzymes and TSH on pain, a multiple linear regression analysis was performed. The regression model included AST, ALT, GGT, and TSH as independent variables, with total pain as the dependent variable. The model was significant (*p* < 0.01), explaining 42% of the variance in total pain (R^2^ = 0.42). The beta coefficients for each predictor were as follows: AST (β = −0.221, *p* = 0.032), ALT (β = −0.237, *p* = 0.029), GGT (β = −0.335, *p* = 0.008), and TSH (β = −0.298, *p* = 0.014). These results indicate that GGT had the strongest predictive value, followed by TSH, ALT, and AST. Together, these metabolic markers significantly predicted pain intensity in patients with frozen shoulder (see Figure 6).

## 4. Discussion

This study investigated the relationship between pain, functionality, and metabolic variables in patients with adhesive capsulitis of the shoulder.

The results of the current study provide novel insights into the biochemical and functional dynamics of patients with FS. Specifically, the observed negative correlations between liver enzymes (AST, ALT, and GGT) and TSH with total pain and functional disability suggest a complex interplay between hepatic function, thyroid activity, and inflammatory responses in these patients. The negative correlation between AST, ALT, and GGT with total pain (r = −0.532, *p* = 0.034; r = −0.537, *p* = 0.032; r = −0.622, *p* = 0.010, respectively) indicates that lower levels of these liver enzymes are associated with increased pain. These enzymes are typically markers of hepatic inflammation and damage. In the context of capsulitis, lower levels might reflect an altered metabolic state potentially linked to systemic inflammation and altered metabolic demands. GGT, in particular, is involved in glutathione metabolism, a critical pathway for managing oxidative stress and inflammation. Reduced GGT levels might indicate compromised antioxidative capacity, contributing to heightened pain perception through increased oxidative stress and inflammatory pathways. The significant negative correlation between TSH and total pain (r = −0.583, *p* = 0.018) and TSH and functional disability (r = −0.516, *p* = 0.041) further supports the hypothesis of a thyroid-related metabolic dysregulation in patients with FS. TSH levels are typically inversely related to thyroid hormone levels, where lower TSH suggests higher thyroid activity. Elevated T3 and T4 thyroid hormones can enhance metabolic rates, potentially leading to a hypermetabolic state. This state can exacerbate pain perception and functional impairment through mechanisms such as increased inflammatory responses, heightened sensitivity of pain pathways, and altered neuromuscular function.

Contrasting these findings with the current understanding of hypothyroidism’s role in capsulitis presents an intriguing discussion [10]. Traditionally, hypothyroidism is considered a risk factor for FS due to its association with musculoskeletal symptoms, including joint pain and stiffness, and increased risk of fibrosis. Patients with hypothyroidism often exhibit elevated TSH levels and low thyroid hormone levels, leading to a slowed metabolic rate and accumulation of glycosaminoglycans in tissues, contributing to fibrosis and inflammation. However, the findings of this study suggest that a hypermetabolic state associated with lower TSH levels might also be a significant factor in capsulitis. Both hypothyroidism and hyperthyroidism involve altered thyroid function and immune responses, but they affect metabolic processes differently. In hypothyroidism, reduced metabolic activity leads to tissue accumulation and fibrosis, while in a hypermetabolic state, increased metabolic demands and oxidative stress may drive inflammatory responses and pain sensitivity.

Despite these differences, both conditions underscore the importance of thyroid health in musculoskeletal disorders; these results are consistent with the study by Dyrek et al., which highlights that the action of thyroid hormones is necessary to maintain proper bone development, mineralization, and strength [23]. They highlight the necessity of monitoring thyroid function in patients with capsulitis and exploring targeted therapies that address these endocrine and immune dysregulations [24]. This comprehensive approach could improve pain management and functional outcomes in these patients, providing a more subtle understanding of the thyroid-capsulitis connection. Additionally, the disruption of the liver–thyroid axis, which refers to the pathological interactions between these two glands, can result in significant metabolic and endocrine alterations. The liver is crucial in the metabolism of thyroid hormones, particularly in the conversion of thyroxine (T4) to triiodothyronine (T3), the most active form of the hormone. Chronic liver diseases, such as cirrhosis, can reduce the activity of hepatic deiodinases, thereby decreasing T3 levels and altering thyroid hormone balance [25]. Furthermore, thyroid dysfunctions can affect liver function. For example, hyperthyroidism can lead to increased insulin resistance and hepatic steatosis, while hypothyroidism can contribute to dyslipidemia and greater lipid accumulation in the liver [26]. These bidirectional interactions underscore the importance of considering both organs in the diagnosis and treatment not only in metabolic and endocrine diseases but in FS.

These findings align with a study by Park et al., indicating that liver function may impact both the incidence and management of patients with frozen shoulder, suggesting that alterations in liver markers could be associated with a higher risk of developing this debilitating condition [27]. However, they showed elevated levels of liver enzymes such as ALT and AST to be correlated with increased prevalence of frozen shoulder and poorer functional outcomes. These findings suggest that liver health may play a significant role in the pathogenesis and severity of frozen shoulder, opening new avenues for research and clinical management of this condition [27]. Furthermore, these findings are also consistent with a recent study published in 2023 indicating a higher prevalence of frozen shoulder in patients with hypothyroidism [28].

However, no significant results were found with the other metabolites studied. Regarding fasting blood glucose measurement, these findings align with Mertens MG et al.’s study, where no evidence was found between blood glucose levels and patients with adhesive shoulder capsulitis compared to a healthy population [18]. In contrast, a literature review published in 2022 by Struyf F. et al. suggests a relationship between patients with glucose impairment or diabetes and shoulder dysfunctions [29]. Thus, FS seems to be more affected when insulin impairment exists than the level of glucose in the blood as a single assessment [30].

Regarding the lipid profile, we did not find any relationship between pain, functionality, and an altered lipid profile. In contrast, a study published in 2018 asserts that there is a connection between hypercholesterolemia and adhesive shoulder capsulitis [14]. These results are consistent with a study where they found concentrations of total cholesterol, triglycerides, and low-density lipoprotein cholesterol in patients with chronic shoulder pain due to rotator cuff tear [31].

The pathophysiology of frozen shoulder is a multifactorial process [32]. It is necessary to consider all influencing factors in patients in order to address this condition in the most efficient way, aiming to improve patients’ quality of life. The restoration of certain metabolite levels could help alleviate pain and reduce dysfunction.

Our gender-based analysis revealed significant differences in the correlations between metabolic biomarkers and pain in men and women with frozen shoulder (FS). Specifically, GGT levels showed a stronger correlation with pain in men (r = −0.620, *p* = 0.010), while ALT and TSH had more significant associations with pain in women (ALT: r = −0.545, *p* = 0.023; TSH: r = −0.572, *p* = 0.015). These findings suggest that different metabolic and hormonal mechanisms may be at play in men and women, influencing the clinical presentation of FS.

One possible explanation for these gender-specific findings lies in the role of the sex hormone axis. Testosterone, the primary male sex hormone, has been shown to have anti-inflammatory effects by reducing the production of pro-inflammatory cytokines such as tumor necrosis factor-alpha (TNF-α) and interleukin−6 (IL−6) [33].

Studies have found that higher testosterone levels are associated with reduced oxidative stress and lower GGT levels, reflecting improved liver function. This could explain the stronger correlation between GGT and pain in men, as testosterone’s anti-inflammatory properties might help mitigate pain sensitization linked to liver dysfunction [34,35]. In contrast, estrogen, the primary female sex hormone, has a more complex and fluctuating influence on pain and inflammation. While estrogen can have protective effects against inflammation, fluctuations in estrogen levels—such as those seen during the menstrual cycle or menopause—are associated with heightened pain sensitivity and an increase in pro-inflammatory cytokines. Estrogen is also known to modulate liver enzyme activity and thyroid function, which could explain the stronger associations between ALT, TSH, and pain in women [33]. Estrogen fluctuations during menopause, for instance, have been linked to changes in liver metabolism and thyroid hormone regulation, potentially exacerbating pain in women with FS [34,36].

The role of the hypothalamic–pituitary–thyroid (HPT) axis further supports these gender differences. Studies have shown that sex hormones, particularly estrogen and testosterone, influence the regulation of the HPT axis, which controls thyroid hormone production. This interaction may explain why thyroid dysfunction, as indicated by TSH levels, shows a stronger correlation with pain in women. Estrogen’s effect on thyroid regulation could be contributing to the altered pain sensitivity and thyroid dysfunction observed in women with FS [37].

These findings highlight the importance of considering sex hormones when evaluating the metabolic and hormonal contributions to FS. The gender-specific correlations between biomarkers and pain suggest that hormonal regulation plays a key role in the pathophysiology of FS. Further research into the sex hormone axis and its impact on liver and thyroid function could open up new avenues for treatment, particularly for women experiencing hormonal changes such as menopause. Hormonal therapies aimed at balancing estrogen levels could potentially alleviate pain and improve outcomes for women with FS.

### 4.1. Prospective

Further studies with larger patient samples are essential to validate and strengthen the findings of this research. While our study has demonstrated a significant correlation between liver and thyroid biomarkers and the pain and functional impairment in patients with adhesive capsulitis of the shoulder, much remains to be explored in this area. Although previous studies have acknowledged metabolic alterations in patients with FS, the specific role of systemic metabolic dysfunction, particularly involving the liver–thyroid axis, is still not fully understood.

A more comprehensive investigation into the multifactorial nature of pain in FS is warranted. It is becoming increasingly clear that pain in FS is influenced by a combination of metabolic, immune, and endocrine factors, rather than being solely attributed to muscular or biomechanical imbalances. By deepening our understanding of these underlying metabolic pathways, future research may uncover novel therapeutic targets that could lead to more effective treatment strategies for this complex condition.

### 4.2. Limitations

As for the limitations of this study, we had a small sample size, so we conducted this initial study as a pilot to observe possible relationships. It would be interesting to further investigate this topic and conduct studies with a larger sample size to reinforce the results of this pilot study. Another limitation was the limited scientific evidence on this topic, which considerably hindered the execution of the study.

## 5. Conclusions

The interplay between liver enzymes, thyroid function, and pain in patients with FS continues to reveal a complex physiological network, underscoring the significant metabolic contributions to the condition. Our findings highlight the potential role of a hypermetabolic state in exacerbating pain and functional impairment, particularly through elevated liver enzymes and dysregulated thyroid activity. This supports the idea that FS is not solely a musculoskeletal disorder but one deeply rooted in metabolic dysfunction. This challenges the traditionally held view of hypothyroidism as a primary factor and suggests that hypermetabolism may also be a critical driver of the condition’s severity. Both hypermetabolic and hypothyroid states involve significant alterations in the thyroid and immune systems, emphasizing the need for an integrated approach that addresses metabolic, immune, and inflammatory components of FS. Future research should further explore these mechanisms to develop tailored therapeutic strategies targeting both metabolic dysregulation and inflammation in patients with FS. By doing so, clinicians can potentially improve pain management and shoulder functionality in this debilitating condition.

## Figures and Tables

**Table 1:** Demographic data of the sample.

		Women					Men					
	Age (years)	Height (cm)	Weight (kg)	BMI	SPADI	NRS	Age (years)	Height (cm)	Weight (kg)	BMI	SPADI	NRS
Mean	55.23	162.34	66.78	23.05	60.31	7.13	56.12	167.45	72.34	25.06	64.95	6.11
Median	55.12	162.50	67.45	23.05	61.03	7.25	56.34	167.23	72.12	24.95	64.86	6.45
Variance	91.38	65.96	99.60	5.63	100.38	1.99	85.07	61.66	83.11	2.24	138.95	2.63
SD	9.56	8.12	9.98	2.37	10.02	1.41	9.22	7.85	9.12	1.49	11.79	1.62

**Abbreviations:** SD = standard deviation; cm = centimeters; kg = kilograms; BMI: body mass index; SPADI = Shoulder Pain and Disability Index; NRS = numerical rating scale.

## Data Availability

Data are contained within the article.

## Abbreviations

FS = frozen shoulder; CAH = adhesive capsulitis; AST = aspartate transaminase; ALT = alanine transaminase; GGT = gamma glutamyltransferase; TSH = thyroid-stimulating hormone.
